# Implantation of heat treatment Ti6al4v alloys in femoral bone of Wistar rats

**DOI:** 10.1007/s10856-022-06691-2

**Published:** 2022-10-03

**Authors:** Mercedes Paulina Chávez Díaz, Soledad Aguado Henche, Mónica Rubio Yanchuck, Celia Clemente de Arriba, Román Cabrera Sierra, María Lorenza Escudero Rincón, José M. Hallen

**Affiliations:** 1 Centro de Estudios Científicos y Tecnológicos Número 7 Cuauhtémoc (CECyT 7), Ermita Iztapalapa 3241, Sta. María Aztahuacan, Iztapalapa, Ciudad de México, 09570 Mexico; 2 Centro Nacional de Investigaciones Metalúrgicas (CENIM-CSIC). Departamento de Ingeniería de Superficies, Corrosión y Durabilidad, 28040 Madrid, Spain; 3 Departamento de Cirugía, Ciencias Médicas y Sociales. Área Anatomía y Embriología Humana de la Facultad de Medicina, Universidad de Alcalá (UAH), Ctra. Mad-Barc Km 33,600. Campus Universitario, Alcalá de Henares, 28805 Madrid, Spain; 4 Hospital Universitario La Paz. Servicio de Cirugía Plástica, Reparadora y Quemados, Paseo de la Castellana 261, 28046 Madrid, Spain; 5 Departamento de Ingeniería Química Industrial y Metalurgia y Materiales, UPALM Edificio 7, Instituto Politécnico Nacional, Ciudad de México, 07738 Mexico

## Abstract

Two heat treatments were carried out at below (Ti6Al4V_800_) and above (Ti6Al4V_1050_) the beta-phase transformation temperature (*T*_*TRANSUS*_ = 980 °C), to study the effect of microstructural changes on osseointegration. The alloys were implanted in the femurs of hind legs of Wistar rats for 15, 30, and 60 days. Histology of the femur sections obtained for the first 15 days showed inflammatory tissue surrounding the implants and tissue contraction, which prevented osseointegration in early stages. After 30 days, trabecular bone, reduction of inflammatory tissue around the implants, and osseointegration were observed in Ti6Al4V as received and Ti6Al4V_1050_ alloys, while osseointegration was detected for the three alloys after 60 days. These results were supported through morphometric studies based on the analysis of Bone Implant Contact (BIC), where there was a larger bone contact after 60 days for the Ti6Al4V_1050_ alloy; indicating that microstructural features of the Ti6Al4V alloys influence their osseointegration, with the lamellar microstructure (Ti6Al4V_1050_), being the most responsive.

**Figure:**
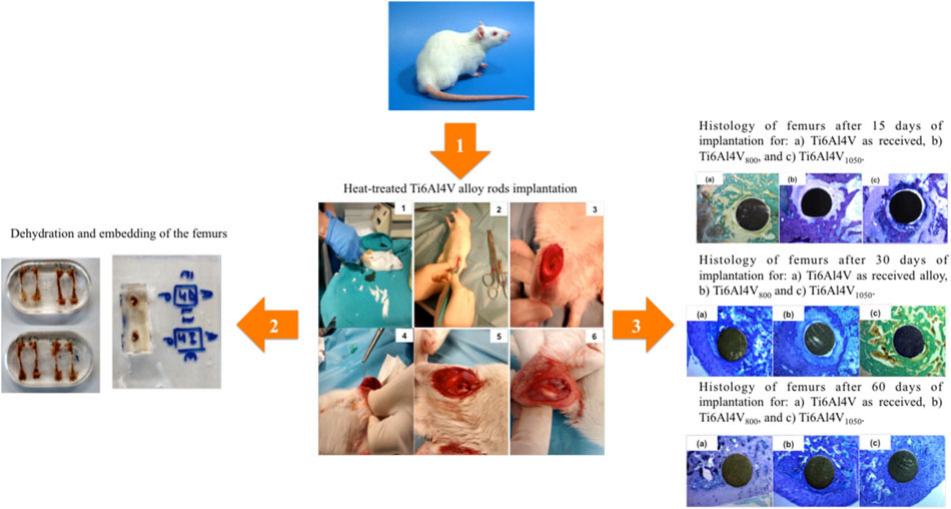
Graphical abstract

Graphical abstract

## Introduction

The study of biomaterials has increased during the last decades; titanium and its alloys have been widely used to manufacture implants (mainly orthopedic) and medical devices due to their corrosion resistance and biocompatibility [1–3]. This is a crucial aspect of biomaterials since the human body’s acceptance of implants depends on it, or rejection [4–6]. Commercially, pure Titanium (CP–Ti) and its alloys present high biocompatibility compared to CoCr alloys and 316L Stainless Steel [1, 7]. Particularly, the Ti6Al4V alloy has been the most widely used since it displays better biological response than CP–Ti [8, 9], not only due to the alloying elements, mainly aluminum, but also its passivation. However, elements such as Al and V have been reported as toxic [10–14]. To solve this problem, several studies have been directed to the design and study of surface treatments and coatings that inhibit or reduce the effect of these elements [7, 13, 15–21]. Currently, studies on heat treatments that modify the surface and bulk properties of the metal are being carried out, and a few works have focused on studying and designing these properties [12, 13]. Heat treatments are mainly aimed at modifying the microstructure of the metals and alloys, which presents an important effect on the interaction with the biological medium and its mechanical behavior [1, 2, 22]. Thus, titanium-based alpha-beta alloys can be subjected to heat treatments below and above their *T*_*TRANSUS*_. Depending on this temperature and the cooling rate, globular, martensitic, lamellar, and bimodal type microstructures can be obtained [23]. These different microstructures will improve or not the behavior of titanium alloys both biologically and mechanically [24, 25].

Bones play an important role in the production of red-white blood cells, platelets, and the storage of minerals such as calcium, magnesium, and phosphorus. Hence, bone tissue is continually changing, being restructured and biologically remodeled throughout life [26]. Biocompatibility is also affected by microstructural changes because the chemical composition of the passive oxide formed on the metal surface is different affecting cell adhesion, biocompatibility, and, consequently, osseointegration [4, 27–29]. Some studies on the performance of Ti and titanium alloys against real biological environments have been carried out through in vivo tests with experimental animals [30–35]. Among these works, it has been reported that there is no bone formation after 30 days of implantation of Ti6Al4V heat-treated alloys in femurs rats [34]. On the contrary, TiO_2_ nanotubes were electrochemically grown on CP-Ti surface and after 6 weeks of implantation a bone/implant interface was observed, supporting the formation of new bone [35]. Based on these results there is lacking information related with the biocompatibility of heat-treated Ti6Al4V alloys. Thus, the aim of this work is studying osseointegration in Wistar rats at 15, 30, and 60 days, of the Ti6Al4V alloy heat treated below (at 800 °C) and above (at 1050 °C) T_TRANSUS_, which provides different microstructures in the alloy.

## Materials and methods

Fifteen Ti6Al4V alloy rods (Goodfellow Ltd) of 1 mm diameter and 15 mm length were used. The rods were sterilized in an autoclave for 30 min at 120 °C and 1.2 Kg cm^–2^. Fifteen 2- to 3-month-old female Wistar rats weighing 200 to 220 g (CHARLES RIVER LABORATORIES France) were used as biological materials. The rods were encapsulated in quartz containing an Argon atmosphere to avoid oxidation. Two heat treatments at 800 and 1050 °C were carried out in the furnace for 11 min and air-cooled at room temperature (normalized); these alloys were named as Ti6Al4V_800_ and Ti6Al4V_1050_, respectively. The implantation of the alloys was performed following the experimental design shown in Table 1. Rats were listed depending on the implantation time: R1–R5, R6–R10, and R11–R15 for 15, 30, and 60 days, respectively. The control rats described as R1, R6, and R11 did not carry implantation of material.

**Table 1:** Design of experiments of Ti6Al4V, Ti6Al4V_800_, and Ti6Al4V_1050_ implantation

Specimen	Right Femur	Left Femur	Implantation time (days)
R1	Control	Control	15
R2	Ti6Al4V_800_	Ti6Al4V_1050_	
R3	Ti6Al4V_800_	Ti6Al4V_1050_	
R4	Ti6Al4V_800_	Ti6Al4V	
R5	Ti6Al4V_1050_	Ti6Al4V	
R6	Control	Control	30
R7	Ti6Al4V_800_	Ti6Al4V_1050_	
R8	Ti6Al4V_800_	Ti6Al4V_1050_	
R9	Ti6Al4V_800_	Ti6Al4V	
R10	Ti6Al4V_1050_	Ti6Al4V	
R11	Control	Control	60
R12	Ti6Al4V_800_	Ti6Al4V_1050_	
R13	Ti6Al4V_800_	Ti6Al4V_1050_	
R14	Ti6Al4V_800_	Ti6Al4V	
R15	Ti6Al4V_1050_	Ti6Al4V	

The implantation was performed in the right and left femurs of the hind legs from the knee. It was selected 15, 30, and 60 days as implantation in order to observe and detect changes due to microstructure around Ti6Al4V implants for early, middle and late stages. After completion of the permeation and/or implantation times, the rats were euthanized by intraperitoneal injection diluted in serum of 0.4 mg sodium pentobarbital (Dolethal^®^). The femurs were extracted, cleaned of muscle tissue, and preserved in flasks with 66 ml of 4% formalin solution for 15 days for fixation; thereafter, dehydration, and inclusion of the femurs were carried out using solutions of GMA (2-Hydroxyethylmethacrylate, Aldrich® Chemistry) with different concentrations to finish in a pure solution of Technovit® (Technovit 7200 VLC, Kulzer), subsequently hardened by photopolymerization. Finally, 50 μm thick cross-sections were cut with an Exakt 312 diamond band saw and polished with an Exakt 400 CS machine (Exakt, Norderstedt, Germany).

For the observation and study of osseointegration, histological staining with toluidine blue and Masson’s trichrome was performed on the cross sections obtained. A variant used for staining incorporates the use of Weigert’s hematoxylin and the subsequent staining with toluidine blue at pH 4. The use of hematoxylin increases the effect of toluidine so that the cell nuclei appear dark blue, almost black. The intercellular substance is clearly differentiated in various shades of blue, depending on the degree of bone maturity. Masson’s trichrome makes it possible to observe osteoid (non-calcified) tissue, which is stained orange, as opposed to mature (calcified) bone, which is seen in green. Cell nuclei are stained dark red. All staining was carried out in a stain-rinse-dry series for 30 min. After obtaining the histological sections, the osseointegration process was quantitatively assessed by capturing the image and transferring it to a computer where the MIP-4 measurement software was used. Osseointegration was assessed by means of the percentage of osseointegration or B.I.C. (Bone Implant Contact), which relates the total perimeter of the implant to the perimeter in direct contact with the bone. The quantitative results were processed using Statgraphics plus 5.1 statistical packages. The significance of the differences between the groups was studied according to the Student’s *t*-test, the one-way Anova test (analysis of variance), where the *P*-value was 0.05.

## Results

### Heat treatments

**Fig. 1:**
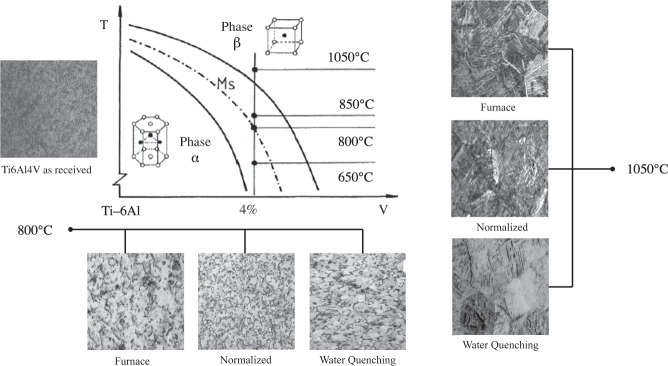
Ti6Al4V alloy phase diagram [27, 28, 39, 40]

The microstructure of the as-received Ti6Al4V alloy consists of equiaxial beta-phase globular particles contained in an alpha-phase matrix (Fig. 1); a similar microstructure with beta-phase dispersed in an alpha-phase matrix was obtained for Ti6Al4V alloy heat-treated at 800 °C. Meanwhile, after the heat treatment at 1050 °C, the microstructure is composed of thin films, where the alpha phase nucleates and diffuses across the grain boundaries of the beta phase (Fig. 1), forming intercalate thin plates of acicular alpha phase, and beta phase [22, 23, 26, 27]. It is important to note that these Ti6Al4V alloys present different microstructural features, which were implanted at different times in Wistar rats (in vivo tests).

### Histology

**Fig. 2:**
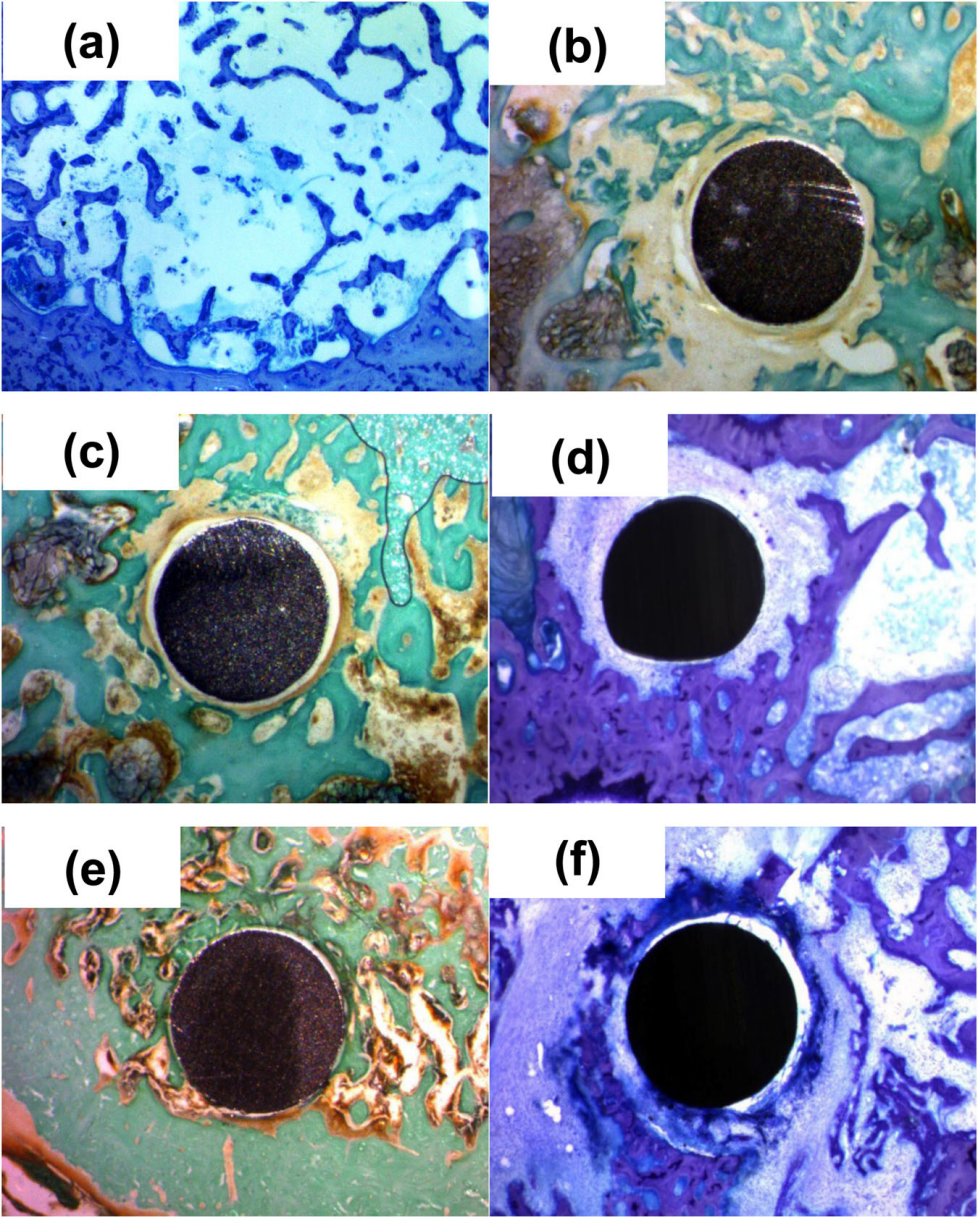
Histology after 15 days of implantation for: **a** control femur **b** Ti6Al4V as received, **c** and **d** Ti6Al4V_800_, **e** and **f** Ti6Al4V_1050_

Figure 2 shows the histological sections obtained after 15 days of implantation. Figure 2a exhibits the control femur (without implant), stained using toluidine blue, whose aspect is normal and corresponds to a diaphysis area, but close to the metaphysis. In the lower part of the image (periphery), an area of the bone cortex is stained blue and forms a thick layer. In the center, there are white areas corresponding to bone marrow, and inside small trabecular bones isolated and/or connected to each other (in blue) is observed. Figure 2b presents the Ti6Al4V alloy as received, stained using Masson’s trichrome, which stains the bone tissue in green. The image shows the implant surrounded by some trabecular bone but separated from it by a line of fibrous tissue. When there is excessive fibrous tissue around the implant, it is not osseointegrated and ends up being lost. On the left, fat cells are observed, which have been stained dark by implant polishing. However, for Ti6Al4V_800_ alloy (Fig. 2c), stained using Masson’s trichrome, bone tissue is shown in green, and a sheet of fibrous tissue (pink line) is observed around the implant, with a white ring appearing between the layer of fibrous tissue and the surface of the implant. It corresponds to an empty area since the non-osseointegration has allowed some tissue retraction. In addition, in other histological sections, this material is surrounded by multiple inflammatory cells (Fig. 2d), which delay or prevent osseointegration.

For the Ti6Al4V_1050_ alloy (Fig. 2e), bone can be observed in the lower left area of the image corresponding to the cortical area of the compact and thick bone, as well as around the implant in green and trabecular bone, between which bone marrow appears as a reddish-black color. Additionally, in other histological sections, some trabecular bone is perceived in a “sea” of inflammatory tissue surrounding the implant (Fig. 2f). According to these results, it seems the Ti6Al4V_1050_ alloy is better osseointegrated than Ti6Al4V_800_ and Ti6Al4V as received during the first 15 days of implantation.

**Fig. 3:**
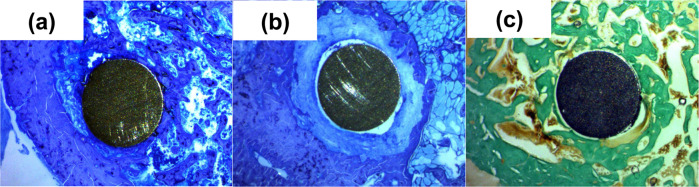
Histology of femurs after 30 days of implantation for: **a** Ti6Al4V as received alloy, **b** Ti6Al4V_800_, and **c** Ti6Al4V_1050_

Figure 3 shows the femur with the implanted materials after 30 days. Figure 3a shows the femur with the Ti6Al4V alloy as received, where cortical bone can be seen on the left of the image and medullary bone on the right. The implant, located in the medullary cavity, is observed rubbing against the endosteum of the nearby cortical bone; besides, trabecular bone can be seen surrounding the implant, indicating the osseointegration of this alloy. For the Ti6Al4V_800_ alloy (Fig. 3b), a thick sheet of fibrous tissue appears around the implant, indicating that there is no osseointegration for this material, likely due to a peri-implant inflammatory process. Meanwhile, for the Ti6Al4V_1050_ alloy (Fig. 3c), trabecular bone is observed around the implant, with some small areas of fibrous tissue (bottom right). The latter shows that Ti6Al4V as received and Ti6Al4V_1050_ alloys are better osseointegrated compared to the Ti6Al4V_800_ alloy. It is important to note the microstructural changes due to heat treatment in the Ti6Al4V_1050_ alloy (lamellar microstructure) compared to the globular microstructure of the Ti6Al4V as received and Ti6Al4V_800_ alloys. Although the last two have a similar microstructure, again it is observed that the Ti6Al4V_800_ alloy is not osseointegrated.

**Fig. 4:**
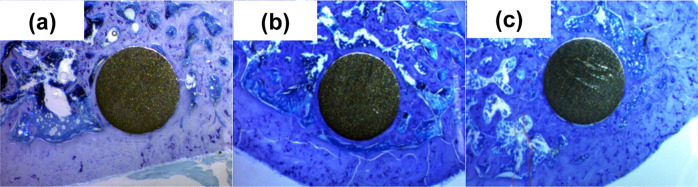
Histology of femurs after 60 days of implantation for: **a** Ti6Al4V as received, **b** Ti6Al4V_800_, and **c** Ti6Al4V_1050_

Figure 4 shows the implants after 60 days. Figure 4a exhibits the Ti6Al4V as received alloy, where the cortical bone is seen in the lower part with some areas with greater staining and vermicular aspect, corresponding to active remodeling processes. The implant is surrounded by bone and a dense trabecular bone from the cortical endosteum can also be seen on the left and right edge of the implant. A similar response is observed for Ti6Al4V_800_ and Ti6Al4V_1050_ alloys (Fig. 4b, c). In both, the implants are in contact with the cortex from which the trabecular bone arises and almost completely surrounds the implant. The thickness of the bone ring surrounding the Ti6Al4V_1050_ implant is thicker indicating that the Ti6Al4V_1050_ alloy has a good osseointegration after 60 days of implantation.

### Morphometry

**Fig. 5:**
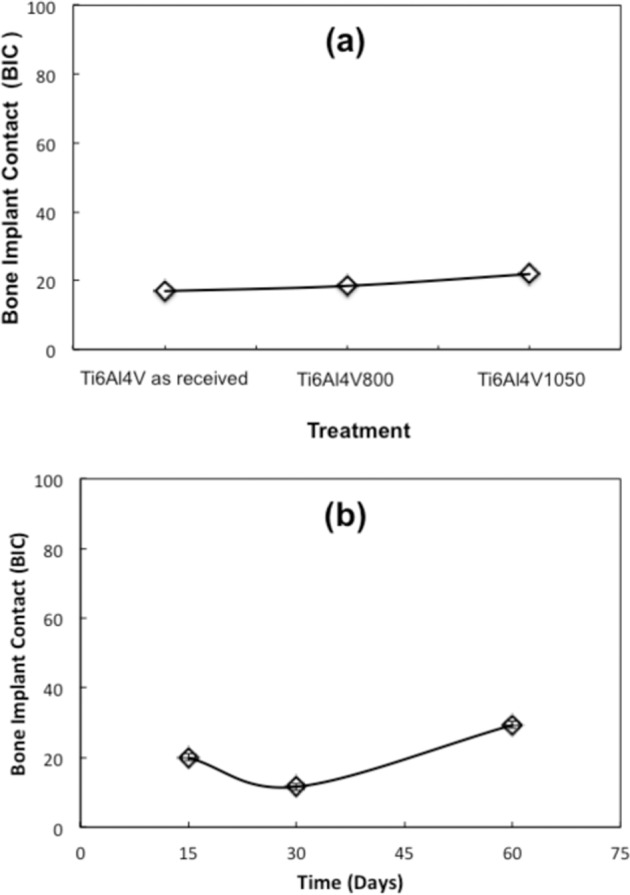
Bone Implant Contact (BIC) for **a** Ti6Al4V as received, Ti6Al4V_800_, and Ti6Al4V_1050_ after 60 days of implanted time and **b** for each study time

The morphometric results obtained for the Bone Implant Contact (BIC) for the three alloys and three implanted times in the femurs of the experimental animals are shown in Fig. 5. Figure 5a shows the BIC for the three alloys, where each point represents the average percentage achieved during the three implantation times. It can be seen the BIC for the Ti6Al4V_1050_ is larger than Ti6Al4V as received and Ti6Al4V_800_ alloys. This indicates that the Ti6Al4V_1050_ alloy presents a greater contact area with the formed bone, associated with a better response in the biocompatibility of the material, compared to the other alloys. This agrees with what was observed in histological studies, that is, as the residence time is longer, better contact is observed between the bone that is being formed and the implant. On the other hand, in Fig. 5b, the BIC is shown for the three implantation times, where each point represents the average percentage achieved for the three alloys at each of the times. It can be observed that after 30 days of implantation is not significantly conclusive, because the BIC at 30 days decreases and then has a tendency to increase at longer times, which is associated with the minimum bone contact in the Ti6Al4V as received and Ti6Al4V_800_ alloys, as showed at the histological study for 15 and 30 days (Figs. 2,3). However, for the Ti6Al4V_1050_ alloy, it was possible to see the bone contact corresponding to the compact cortical and trabecular bones. The Ti6Al4V as received alloy shows a tendency to remain constant at longer implantation times (Figs. 3, 4), while the Ti6Al4V_800_ alloy generates minimum bone contact; this latter due to the fibrous tissue which is still observed around the implant due to inflammatory processes. After 60 days, the three alloys are observed to have bone contact, with dense trabecular bone coming from the cortical endosteum (higher for the Ti6Al4V_1050_ alloy).

## Discussion

In this study, Ti6Al4V alloy rods were heat-treated at 800 and 1050 °C. In addition to these alloys, Ti6Al4V as received were implanted in the right and left femurs of the hind legs from the knee of 15 female Wistar rats.

Based on histology studies, inflammation around the implants and tissue shrinkage were observed after 15 days of implantation, which prevented osseointegration in early stages, except for the Ti6Al4V_1050_ alloy. After 30 days, a decrease in inflammation and trabecular bone formation was observed around the Ti6Al4V as received and Ti6Al4V_1050_ implants. Osseointegration is observed in the three materials only at 60 days of implantation. These results are important to mention, because Nyoman Jujur et al., performed heat treatments at 800 and 1050 °C using the Ti6Al4V alloy (ELI), which were inserted for 30 days in femurs of Sprague Dawley rats, reporting that heat treatments do not affect the level of bone regeneration and maturation [34]. On the contrary, in another work reported by Yi Young-Ah et al., the titanium surface was anodized to produce TiO_2_ nanotubes with different diameters. The materials were implanted in the femurs of fifty male Sprangue Dawley rats for a period of 2 and 6 weeks, where it has been observed that after 6 weeks of implantation the formation of new bone continues with better osseointegration on the surface of the alloys. and greater bone mineralization has been detected. After 12 weeks it has been possible to see bone tissue remodeling and hard tissue surrounded by bone marrow containing collagen fibers, vessels, adipocytes, and leukocytes, which indicates that the formation of new bone takes more than 4 weeks (30 days) [35].

Assuming that the differences are minimized by controlling for weight, age, sex, and randomization, these results are consistent with those observed in the osseointegration of the Ti6Al4V as received, Ti6Al4V_800_, and Ti6Al4V_1050_ alloys. According to these results, the Ti6Al4V_1050_ alloy exhibited a better osseointegration after 60 days, this can be related to its lamellar microstructure, compared to the globular microstructure of the Ti6Al4V as received and Ti6Al4V_800_ alloys. In these Ti6Al4V alloys, the alpha phase is thickened and surrounds the beta phase, that is, the beta phase grains are immersed in an alpha phase matrix; while in the Ti6Al4V_1050_ alloy the beta phase is retained and/or trapped within the alpha phase. This fact indicates that the microstructure plays an important role but especially in the passive titanium oxide composition. It is likely to consider a strong interaction between the passive behavior of the titanium oxide on the surface and the biological medium. In a previous work studying the passivation of the Ti6Al4V alloy in a Hank solution at 37 °C, imposing different oxidation potentials and using the XPS technique, the formation of a film composed mainly of TiO_2_, highly defective and partially hydrated (formation of an oxyhydroxide) has been reported. In this work, the passive behavior was characterized by the EIS technique and is due to the mobility of oxygen and hydroxyl vacancies in the TiO_2_ matrix which could affect the behavior of this oxide and to facility the adsorption of oxygen species such as OH^–^, O^2–^, PO_4_^3–^ or H_2_O, affecting cell adhesion, biocompatibility and, consequently, osseointegration [36, 37].

These results can be related to those obtained in previous works by the research group on the adhesion and proliferation of bone cells, which allowed selecting the Ti6Al4V_800_ and Ti6Al4V_1050_ alloys (normalized) to perform the in vivo tests. The most relevant results of the in vitro biological assays carried out on osteoblast and fibroblast cells after 7 days of immersion are summarized in Table 2 [26, 27]. In the presence of osteoblast cells, a higher amount of Ca was detected by EDS for the normalized Ti6Al4V_1050_ alloy, which is related to higher hydration of the surface. The analysis by XPS revealed the presence of Ca and P for this alloy. A lower cell adhesion was detected on Ti6Al4V as received and Ti6Al4V_800_ alloys, so the cell response was better on the Ti6Al4V_1050_ alloy.

**Table 2:** Summary results [26, 27]

Specimen	Microstructure	EDX osteoblasts	Osteoblast Morphology	EDX fibroblasts	Fibroblast morphology
Ti6Al4V	Globular	Ca and P were not detected	Polygonal elongated	Ca was not detected P 0.61%w S 0.41%w	Polygonal elongated and round
Ti6Al4V_800_	Globular	Ca 0.11%w P 0.28%w	Polygonal elongated	Ca was not detected P 0.43%w S 0.26%w	Polygonal elongated and round
Ti6Al4V_1050_	Lamellar	Ca 0.33%w and P was not detected	Polygonal and round and larger coating	Ca 0.68%w P 0.47%w S 0.31%w	Polygonal elongated and round. Larger coating

In the presence of fibroblast cells, Ca and P elements are important for mineralization and they were only detected for the Ti6Al4V_1050_ alloy (Table 2). An important point to highlight is the presence of S in the three alloys, whose proportion is higher on the Ti6Al4V as received alloy (Table 2). This element could be inhibiting and/or preventing the anchorage of Ca because only when the proportion of S decreases P is detected, both elements maintain a proportional S/P ratio. This ratio is larger for the Ti6Al4V_1050_ alloy, on which Ca is detected (Table 2). As observed in previous works, Ti6Al4V_1050_ alloy shows better biological response, i.e., osteoblast and fibroblasts cell adhesion and proliferation, which has been related to its lamellar-like microstructure. These findings could be related to the fact that the biological response in this alloy is better and that bone maturation occurs in earlier stages, i.e., from the first 15 days. Furthermore, the effect of the microstructure generated from the thermal treatment, influences the growth of the passive oxide [26] whose chemical composition will allow or not the osseointegration through bone formation. As has been seen in the in vivo tests, the osseointegration is slower in the Ti6Al4V as received and Ti6Al4V_800_ alloys.

In conclusion, changes in the microstructure, and especially on lamellar-like, can improve the biological response and subsequently facilitate the osseointegration; as observed in the histology study, Ti6Al4V_1050_ alloy shows better osseointegration after 60 days of implantation and could be used as implant material. Meanwhile, we need to explore if other microstructures, such as bimodal-like one, can improve cell adhesion and proliferation and subsequently its biocompatibility and osseointegration [38].

## Conclusions

Ti6Al4V as received and Ti6Al4V_800_ alloys both have globular type microstructures whereas Ti6Al4V_1050_ has lamellar type microstructure, but respond differently. The histological study after 15 days of implantation showed peri-implant trabecular bone with no differences between three treated groups. In addition, inflammatory cells were observed. At 30 days of implantation, a thick ring of fibrous tissue was observed around the Ti6Al4V_800_ alloy implant associated with an inflammatory process. Moreover, bone trabeculae were observed around the Ti6Al4V_800_ and Ti6Al4V_1050_ alloys after 30 and 60 days of implantation, associated to biocompatibility and osseointegration, which is related to microstructure features. Ti6Al4V_1050_ alloy has the best behavior in terms of biocompatibility and osseointegration because of a better bone tissue contact with the alloy in earlier stages.
